# *PSMB9* Codon 60 Polymorphisms Have No Impact on the Activity of the Immunoproteasome Catalytic Subunit B1i Expressed in Multiple Types of Solid Cancer

**DOI:** 10.1371/journal.pone.0073732

**Published:** 2013-09-09

**Authors:** Ji Eun Park, Lin Ao, Zachary Miller, Kyungbo Kim, Ying Wu, Eun Ryoung Jang, Eun Young Lee, Kyung Bo Kim, Wooin Lee

**Affiliations:** 1 Department of Pharmaceutical Sciences, College of Pharmacy, University of Kentucky, Lexington, Kentucky, United States of America; 2 Department of Pathology and Laboratory Medicine, College of Medicine, University of Kentucky, Lexington, Kentucky, United States of America; Vanderbilt University Medical Center, United States of America

## Abstract

The proteasome is a key regulator of cellular protein homeostasis and is a clinically validated anticancer target. The immunoproteasome, a subtype of proteasome expressed mainly in hematopoietic cells, was initially recognized for its role in antigen presentation during the immune response. Recently, the immunoproteasome has been implicated in several disease conditions including cancer and autoimmune disorders, but many of the factors contributing to these pathological processes remain unknown. In particular, the codon 60 polymorphism of the *PSMB9* gene encoding the β1i immunoproteasome catalytic subunit has been investigated in the context of a variety of diseases. Despite this, previous studies have so far reported inconsistent findings regarding the impact of this polymorphism on proteasome activity. Thus, we set out to investigate the impact of the *PSMB9* codon 60 polymorphism on the expression and activity of the β1i immunoproteasome subunit in a panel of human cancer cell lines. The β1i-selective fluorogenic substrate Acetyl-Pro-Ala-Leu-7-amino-4-methylcoumarin was used to specifically measure β1i catalytic activity. Our results indicate that the codon 60 Arg/His polymorphism does not significantly alter the expression and activity of β1i among the cell lines tested. Additionally, we also examined the expression of β1i in clinical samples from colon and pancreatic cancer patients. Our immunohistochemical analyses showed that ∼70% of clinical colon cancer samples and ∼53% of pancreatic cancer samples have detectable β1i expression. Taken together, our results indicate that the β1i subunit of the immunoproteasome is frequently expressed in colon and pancreatic cancers but that the codon 60 genetic variants of β1i display similar catalytic activities and are unlikely to contribute to the significant inter-cell-line and inter-individual variabilities in the immunoproteasome activity.

## Introduction

The proteasome is responsible for the degradation of targeted proteins and is a key player in the maintenance of cellular protein homeostasis and the regulation of cellular processes that are essential in cancer development and progression [1], [2]. The immunoproteasome, an alternative form of the proteasome, is found in cells of hematopoietic origin, but its expression can also be induced under inflammatory and stress conditions in other cell types [3]. The immunoproteasome is formed when the three types of catalytic subunit found in the constitutive proteasome, β1 (Y, *PSMB6*), β2 (Z, *PSMB7*) and β5 (X, *PSMB5*), are replaced by three homologous immuno-subunits: β1i (LMP2, *PSMB9*), β2i (MECL-1, *PSMB10*) and β5i (LMP7, *PSMB8*). Compared to the constitutive proteasome, the immunoproteasome is found to have slightly altered proteolytic specificities which are capable of producing peptides more suitable for binding to the major histocompatibility complex I molecules thus facilitating antigen presentation [4]. However, recent studies indicate that the immunoproteasome may have important functions beyond the adaptive immune response. For instance, the immunoproteasome is found to be expressed in non-inflammed, immune-previleged tissues (e.g. retina and brain [5], [6]) and in the context of several disease states (e.g. cancer, neurodegenerative diseases, autoimmune diseases, [3], [7] and references therein). Despite these observations, the biological significance of the immunoproteasome in such disease states is not fully understood.

The β1i subunit of the immunoproteasome is encoded by the *PSMB9* gene located on chromosome 6. This gene harbors a commonly occurring genetic R/H polymorphism at codon 60 (p.60R>H; c.179G>A; rs17587) with H allele frequencies of 1.1% to 34%, varying across ethnic groups ([8] and references therein). Several investigations have reported potential associations between codon 60 *PSMB9* polymorphism status and increased susceptibility to various diseases such as insulin-dependent diabetes mellitus, rheumatoid arthritis and multiple sclerosis [8]–[16]. However, it is difficult to decipher from previous studies whether the observed associations are directly related to the altered activity of the β1i subunit as a result of the R/H amino acid variation. This is in part due to the inter-dependent nature of proteasome subunits and the lack of appropriate molecular probes. A previous investigation by Mishto et al. [17] examined more closely at the impact of this polymorphism on proteasome activity in the aged brain using the fluorogenic peptide substrate N-Succinyl-Leu-Leu-Val-Tyr-AMC (Suc-LLVY-AMC). The results from this study suggested that the H allele results in a decreased proteasome activity in the aged brain [17]. However, since Suc-LLVY-AMC is conventionally used to measure the overall chymotrypsin-like (CT-L) proteolytic activity of the proteasome and thus can be hydrolyzed by multiple subunits of both the immunoproteasome and the constitutive proteasome, this decrease in the hydrolysis of Suc-LLVY-AMC may not necessarily indicate changes in β1i function. Furthermore, a subsequent study by the same group using recombinant peptides mimicking endogenous substrates indicated no differences in the substrate hydrolysis profiles between the codon 60 genotypes [18].

Much of the discrepancy regarding the functional impact of the PSMB9 codon 60 polymorphism arises from the lack of a tool to specifically observe the function of the β1i subunit. In this regard, Lin et al. [19] and Blackburn et al. [20] recently reported on the development and application of the fluorogenic substrate Acetyl-Pro-Ala-Leu-7-amino-4-methylcoumarin (Ac-PAL-AMC) which is hydrolyzed selectively by β1i. This novel tool has made it possible to assess the direct functional impact of *PSMB9* codon 60 polymorphism on the β1i subunit. In our current study, we investigated the expression of β1i in clinical colon and pancreatic cancer tissues as well as in established human cancer cell lines. Using the β1i-selective fluorogenic substrate Ac-PAL-AMC, we also assessed the catalytic activity of β1i in multiple cancer cell lines carrying different genotypes at codon 60. Our results indicated that β1i is frequently expressed in colon and pancreatic cancers, but the codon 60 *PSMB9* polymorphism has no significant impact on the catalytic activity of β1i expressed in multiple types of cancer cell lines.

## Materials and Methods

### Cell Culture and Reagents

Established cell lines derived from various types of human cancers (colon, HCT-8, DLD-1, HCT-116, SW480; lung, H23, H358, H460, H727, H1299; prostate, PC-3, DU145; pancreas, BxPC-3, PANC-1, AsPC1; breast, MCF-7, HS578T, MDA-MB-231) were purchased from the American Type Culture Collection (ATCC) and maintained under the recommended conditions. The fluorogenic substrates Suc-LLVY-AMC and Ac-PAL-AMC were custom-synthesized following the standard peptide synthesis strategy. Proteasome inhibitors UK-101 and epoxomicin were synthesized and purified as reported previously [21], [22]. Purified 20 S constitutive proteasome and immunoproteasome were purchased from Boston Biochem.

### Immunohistochemical Analysis

Tissue microarrays containing de-identified, archival cases of human colon and pancreatic cancer tissue specimens were obtained from US Biomax. The use of de-identified tissue array samples from a commercial source for the current study was deemed to be exempt from the Human Subject Regulation by the Institutional Review Board. A streptavidin-biotin-immunoperoxidase assay was performed after the antigen retrieval procedure (citrate buffer, pH 6) using a monoclonal antibody against β1i (1∶100 dilution, Enzo Life Sciences) according to the previously reported protocol [23]. Immune reaction was visualized using 3,3′-diaminobenzidine (DAB), and nuclei were counterstained with hematoxylin. The specificity of immunoreactive signals was verified by omitting either the primary or the secondary antibody. The immunostained tissue microarray sections were analyzed by an experienced pathologist (Dr. Eun Y. Lee). The intensity of immunostaining was assigned on a scale of 0 to 2 or 3.

### Determination of *PSMB9* Polymorphic Status at Codon 60

The *PSMB9* genotypes at codon 60 were initially determined using standard PCR methods and DNA enzymatic digestions as previously reported [24]. The amplicon covering the entire open reading frame was subsequently cloned and analyzed by direct sequencing to verify that the cell lines do not contain additional genetic variations in the PSMB9 gene.

### Immunoblotting Analysis

An equivalent amount of tissue or cell extracts were resolved in polyacrylamide gels and transferred to PVDF membranes. Membranes were blocked in 5% skim milk and probed with anti-β1i (1∶1000, Abcam) and anti-β-actin (1∶1000, Novus) antibodies. After washing, the blots were incubated with horseradish peroxidase-conjugated secondary antibodies. Bound antibodies were detected using an enhanced chemiluminescence substrate (Pierce Biotechnology). In order to compare the relative expression levels of β1i among multiple cell lines, serially diluted H23 cell extracts were used as calibration standards and band intensities were densitometrically quantified by using the Quantity One software (Bio-Rad).

### Proteasome Activity Assays

The catalytic activity of β1i was determined by monitoring the hydrolysis rate of fluorescent 7-amino-4-methylcoumarine (AMC) from Ac-PAL-AMC [20]. Briefly, purified proteasomal preparations or cell extracts containing equivalent total protein amount (10 µg) were added to 96 well plates and adjusted to a final volume of 50 µl using assay buffer (20 mM Tris/Cl, 0.5 mM EDTA, pH 8.0). The reaction was initiated by adding Ac-PAL-AMC (100 µM) and the fluorescence of liberated AMC was monitored for 90 min using a SpectraMax M5 plate reader (Molecular Devices, excitation 360 nm and emission 460 nm). In order to verify that substrate hydrolysis is mediated by β1i, additional sets of cell extracts or purified proteasomal preparations were pre-treated with proteasome inhibitors UK101 or epoxomicin for 1 h, after which fluorescent signals were monitored. In separate experiments, the hydrolysis of Suc-LLVY-AMC (100 µM) was monitored to assess the overall CT-L proteolytic activity.

### Statistical Analysis

Values from data were expressed as means with standard deviations. A comparison between the groups was performed using the Student’s *t*-test and p<0.05 was considered significant.

## Results

### Frequent Expression of β1i in Colon and Pancreatic Cancer Tissues

**Figure 1:**
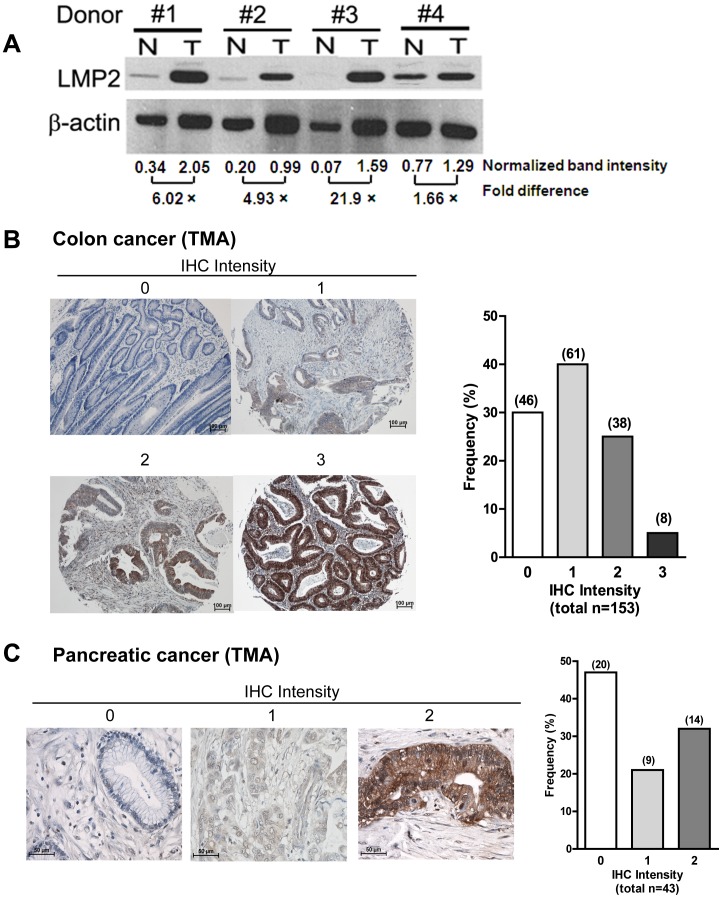
β1i is frequently expressed in colorectal and pancreatic cancer tissues. (a) Immunoblotting analysis for β1i using protein lysates prepared from nonmalignant (N) and cancerous (T) colonic tissues from the same donors (n = 4 pairs). β-actin was used as a loading control. The band intensities for β1i and β-actin were densitometrically analyzed and used to obtain the relative β1i expression normalized to β-actin levels. (b) Immunohistochemical staining for β1i using a tumor array containing 153 evaluable tumor colon tissue specimens. The intensities of β1i positive staining were evaluated on a scale of 0 to 3. Approximately 70% (46 specimens had intensity of grade 1, 38 with grade 2, and 8 with grade 3, out of 153 total tumor specimens) of colorectal tissues have positive (the staining intensity ≥1) β1i staining. (c) Immunohistochemical staining for β1i using a tumor array containing 43 evaluable tumor pancreatic tissue specimens. The intensities of β1i positive staining were evaluated on a scale of 0 to 2. Approximately 53% (9 specimens with intensity grade 1, and 14 with grade 2, out of 43 total tumor specimens) of pancreatic cancer tissues had ≥1 β1i staining intensity.

In assessing the expression of β1i in colon cancer, we first utilized four pairs of colon cancer and nonmalignant adjacent colonic tissues from matching donors. Our immunoblotting analyses indicated that β1i levels were highly elevated in all four clinical colon cancer tissues compared to the paired nonmalignant colonic tissues (Figure 1A). To further evaluate the frequency of β1i expression in colon cancer, we performed immunohistochemical analyses on a tumor array containing 153 evaluable colon cancer specimens of all clinical stages. The intensity of β1i staining was evaluated on a scale of 0 to 3 and our results indicated that the majority (approximately 70%, n = 107 out of 153 total specimens) of colon cancer tissues were positive (the staining intensity ≥1) for β1i staining (Figure 1B). Similar analyses were performed using a tumor array containing 43 evaluable pancreatic cancer specimens of all clinical stages. Due to the limited sample size, the intensity of β1i staining was evaluated on a scale of 0 to 2 and our results indicated that approximately 53% (n = 23 out of 43 total specimens) of pancreatic cancer tissues had positive (the staining intensity ≥1) β1i staining (Figure 1C). For both tissue arrays, we assessed whether there is any association between the β1i expression and available clinicopathologic factors (e.g. gender and tumor grade). However, the results did not reveal any apparent associations.

### Catalytic Activity of β1i in a Panel of Human Cancer Cell Lines Carrying Different Codon 60 Polymorphic Variants

**Figure 2:**
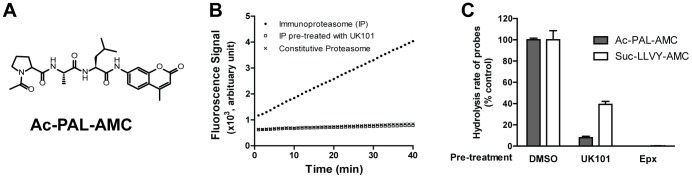
Ac-PAL-AMC is hydrolyzed selectively by β1i. (a) Chemical structure of Ac-PAL-AMC. (b) Ac-PAL-AMC hydrolysis over time by purified constitutive proteasome (cross), purified immunoproteasome (closed circle), or purified immunoproteasome pre-treated with UK101 (open square). A linear increase was seen in immunoproteasome-containing wells only. (c) The rate of hydrolysis of Ac-PAL-AMC or Suc-LLVY-AMC in the presence of Panc-1 cell extract (10 µg). Hydrolysis of Ac-PAL-AMC was almost completely inhibited by UK101 pre-treatment (10 µM). Hydrolysis of Suc-LLVY-AMC was partially inhibited by UK-101 pre-treatment. Epoxomicin (Epx, 10 µM), a broadly-acting proteasome inhibitor, was used as a positive control.

Previously, Blackburn et al. [20] reported that Ac-PAL-AMC (Figure 2A) can be used as a selective probe for measuring the catalytic activity of β1i. Prior to the use of Ac-PAL-AMC in our study, we further verified that the hydrolysis of Ac-PAL-AMC was selectively mediated by β1i using purified preparations of immunoproteasome and constitutive proteasome. Indeed, Ac-PAL-AMC was readily hydrolyzed by the immunoproteasome, but not by the constitutive proteasome (Figure 2B). Pretreatment with UK101, a β1i-selective proteasome inhibitor [21], completely inhibited Ac-PAL-AMC hydrolysis, further validating Ac-PAL-AMC as a β1i-selecitve probe (Figure 2B). Similarly, approximately 90% of hydrolysis of Ac-PAL-AMC was inhibited in Panc-1 cell extracts by UK-101 pre-treatment (Figure 2C). In contrast, the hydrolysis of Suc-LLVY-AMC was only partially blocked by UK101 pretreatment in Panc-1 extracts, suggesting that Suc-LLVY-AMC is hydrolyzed by proteasome subunits other than β1i (i.e. β5 or β5i) (Figure 2C).

**Figure 3:**
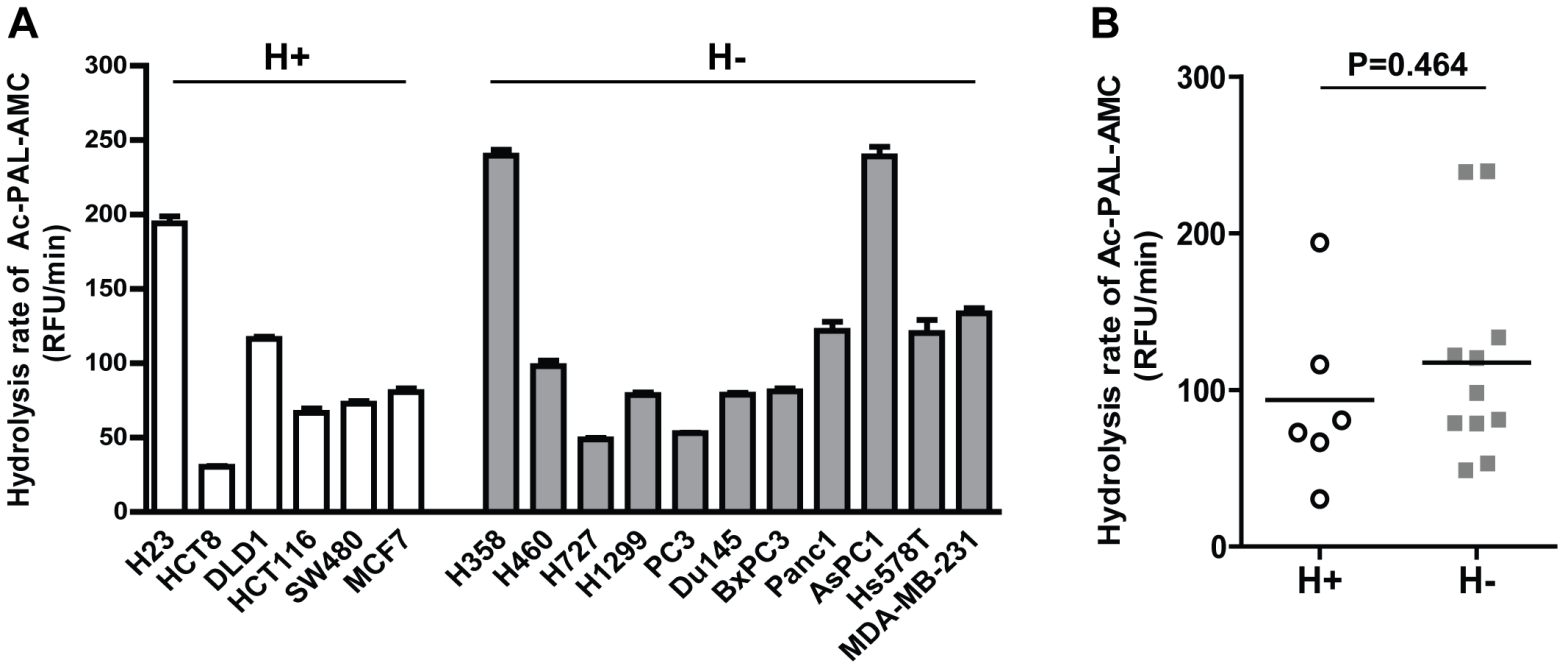
The catalytic activity of β1i is not affected by the *PSMB9* codon 60 polymorphisms. (a) The catalytic activity of β1i measured by rate of hydrolysis of Ac-PAL-AMC in human cancer cell lines with H/H or R/H genotype (blank bars) and R/R genotype (filled bars). Variable activity was observed among cell lines. (b) Rate of Ac-PAL-AMC hydrolysis in cell lines with H/H or R/H genotype (open circles) or R/R genotype (closed squares). Rate of hydrolysis did not show any statistically significant difference between H+ and H- cell lines.

Following the validation of Ac-PAL-AMC as a β1i-selective probe, we then assessed the β1i activity in a panel of human cancer cell lines. These cell lines were screened for their *PSMB9* genotypes at codon 60 (n = 6 for those carrying HH or HR genotype; n = 11 for those carrying RR genotype) and verified not to have any additional variations in the *PSMB9* gene by direct sequencing (Table S1). Our results indicated that the catalytic activities of β1i assessed by the hydrolysis rate of Ac-PAL-AMC substantially vary among these cell lines, however these differences do not seem to be associated with the codon 60 polymorphic status (Figure 3).

### β1i Expression in Human Cancer Cell Lines Carrying Different Codon 60 Polymorphic Variants

**Figure 4:**
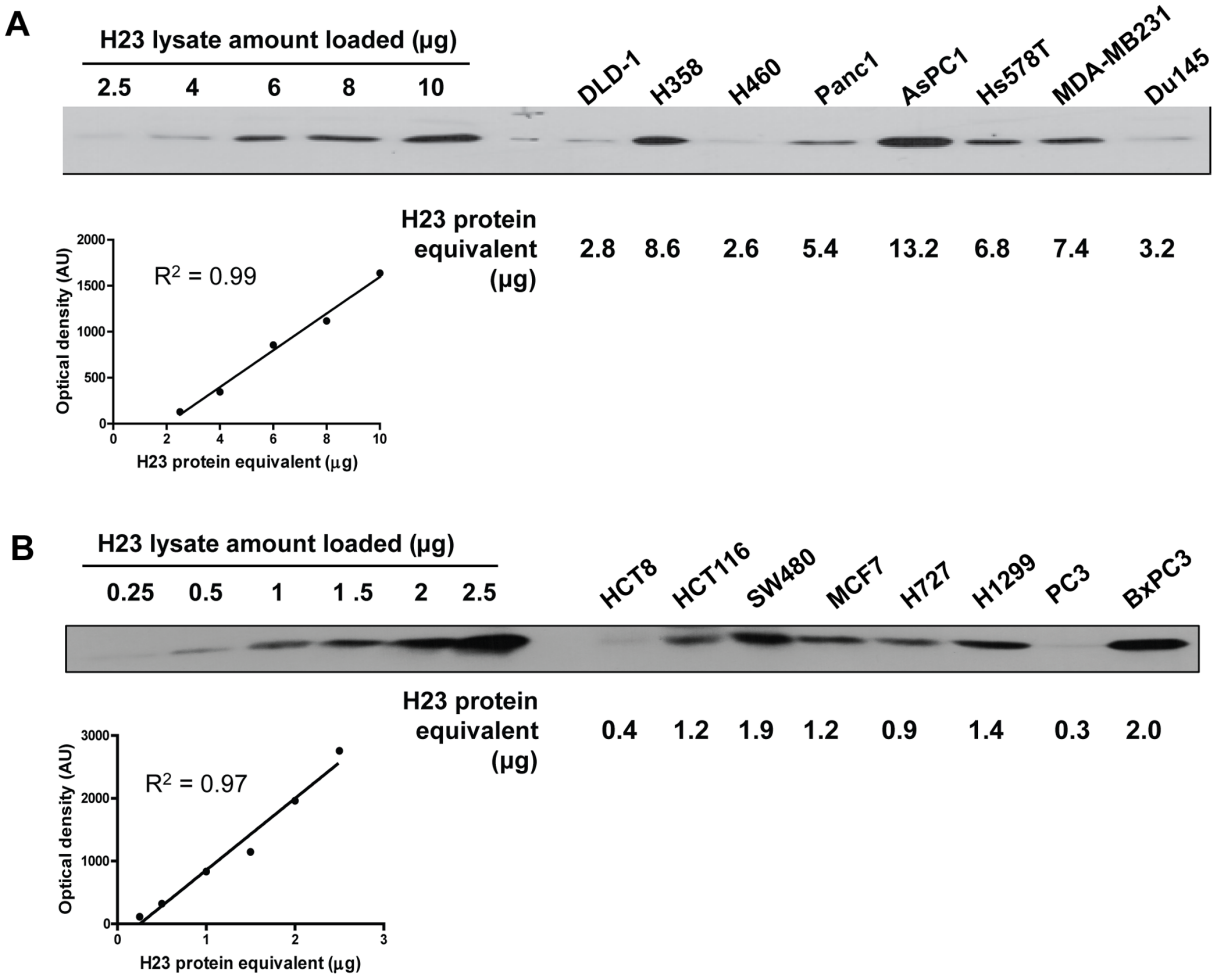
Determination β1i expression levels across multiple human cancer cell lines. An equivalent amount (10 µg) of cell extracts were subjected to immunoblotting with an antibody to β1i along with serially diluted H23 cell extracts (calibration standards). Densitometric analyses of band intensities were performed to quantify relative expression levels of β1i. Cell lines expression high levels of β1i are shown in (A) and cell lines with low expression of β1i are shown in (B).

**Figure 5:**
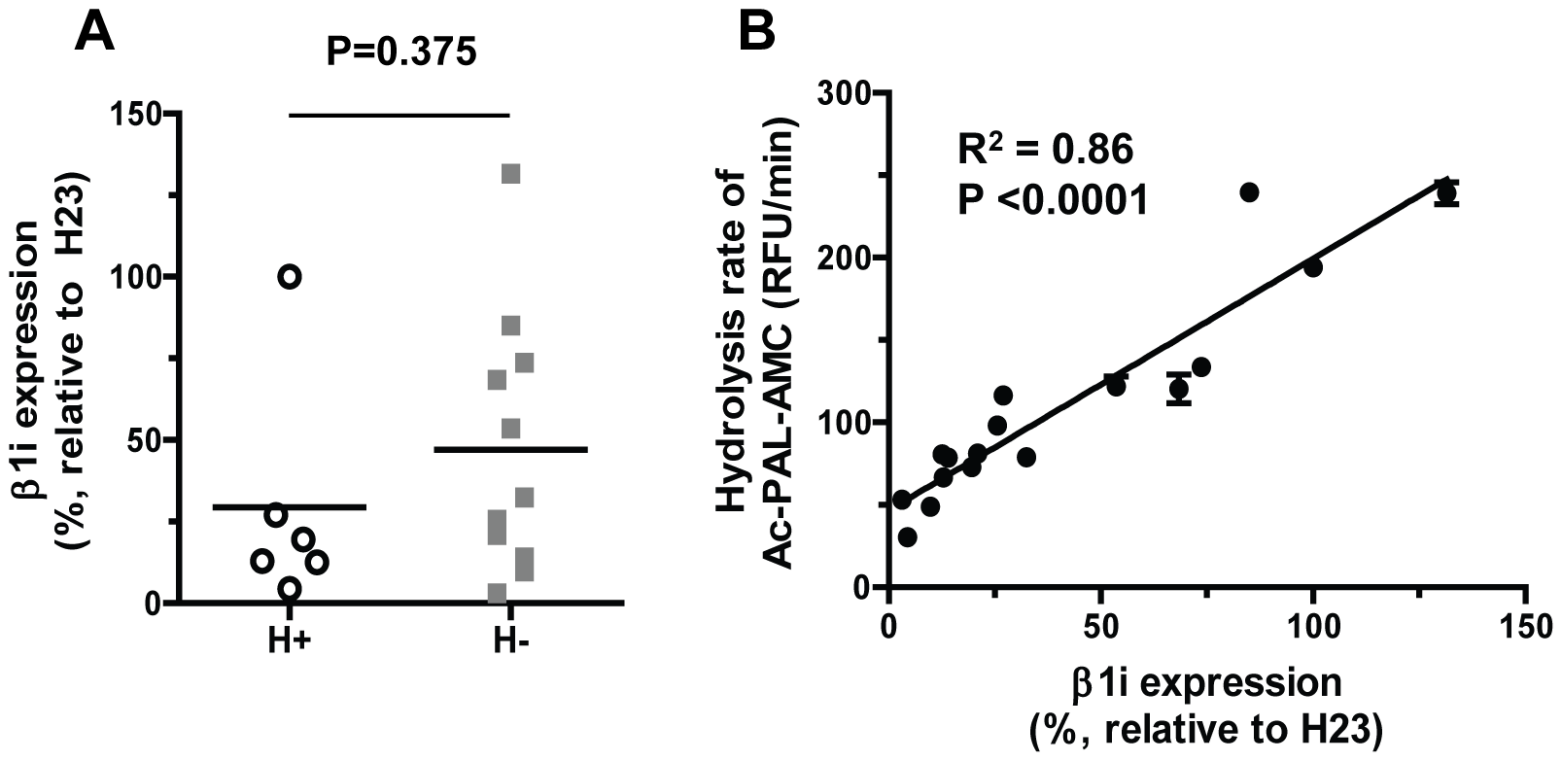
The expression levels of β1i are not affected by the *PSMB9* codon 60 polymorphisms, but associated with the β1i activity. (a) β1i expression in cell lines with H/H or R/H (open circle) and R/R genotypes (closed square). β1i expression did not show statistically significant difference between H+ and H- cell lines. (b) Correlation of Ac-PAL-AMC hydrolysis with β1i expression in all cell lines tested. A highly significant correlation was noted between the expression levels of β1i and its catalytic activity (R^2^ = 0.86, p<0.0001).

As a next step, we examined whether the β1i expression levels are influenced by the codon 60 polymorphic status in the tested cancer cell lines. In order to quantify β1i protein levels, which display substantial variability across different cell lines, we used serially diluted H23 cell extracts as calibration standards (Figure 4). A comparison of cell lines with the H-allele to those that lack the H-allele showed no statistically significant differences in β1i expression (Figure 5A). Instead, we found a highly significant correlation between the catalytic activity β1i and its expression level (Figure 5B, r^2^ = 0.86, p<0.0001, individual values are included in Table S1). These findings suggested that the differing expression levels of β1i likely account for the majority of variability observed in the β1i activity across different cell lines.

## Discussion

In our current study, we investigated the impact of the *PSMB9* codon 60 polymorphisms on the β1i subunit of the immunoproteasome expressed in multiple cancer cell lines using a recently reported β1i activity probe, Ac-PAL-AMC. Our results indicated that the codon 60 polymorphism has no major impact on the expression levels or catalytic activities of β1i in the panel of human cancer cell lines tested. These results are different from previous findings which indicated that brain tissue from aged individuals carrying the codon 60 H allele had decreased proteasome activity compared to tissue from those who did not carry the codon 60 H allele [17]. A possible reason for these apparent discrepancies may be related to the different substrates used to assess proteolytic activity. The fluorogenic substrate Suc-LLVY-AMC used by Mishto et al. [17] is commonly used to measure the overall chymotrypsin-like activity of the proteasome. However, since this substrate can be hydrolyzed by both the immunoproteasome (β1i and β5i) and the constitutive proteasome (β5) [25], the results obtained using Suc-LLVY-AMC cannot tease out the direct contribution of the β1i genetic polymorphism. Our current results were obtained using the recently reported substrate Ac-PAL-AMC that is selectively cleaved by the β1i subunit (Figure 2) [20]. Our findings are consistent with the previous report that the *PSMB9* codon 60 polymorphism had no impact on the degradation profiles of a 28-mer peptide (Kloe 258) and a recombinant form of IkBα, a key regulator of classical NF-κB pathway and well-known proteasome substrate [18]. Although we cannot completely rule out the possibility that the impact of the codon 60 polymorphism may vary depending on the substrate and disease types, the results from the current study and others suggest that the codon 60 polymorphism will not likely contribute to inter-individual variability in β1i catalytic activity levels.

With the remarkable clinical successes of bortezomib and carfilzomib in treating multiple myeloma and other hematological malignancies, the proteasome is now recognized as an important chemotherapeutic target. However, undesirable toxicities limit the broad use of the currently available proteasome-targeting drugs. To overcome these limitations, the immunoproteasome, an alternative form of the constitutive proteasome, has been explored as an alternative therapeutic target. Such efforts have yielded promising immunoproteasome-targeting compounds with anticancer efficacy and improved toxicity profiles [7], [23], [26], [27]. In exploring immunoproteasome-targeting approaches for cancer therapy, it is important to consider the immunoproteasome expression across different cancer types. While the immunoproteasome is known to be upregulated in hematological malignancies, the expression of the immunoproteasome in solid cancer has not been thoroughly examined. In the current study, we report that the β1i subunit is frequently expressed in clinical tissue specimens from colon and pancreatic cancers as well as a panel of human cancer cell lines derived from colon, lung, prostate and breast tissues. Although similar findings have been reported by our group and others [21], [23], [28], [29], our current study provides more robust information regarding the expression of β1i in the majority of clinical colon and pancreatic cancer tissues tested. It should be noted that the downregulation of immunoproteasome subunits has also been reported in some types of cancer [30]–[34] and it is possible that the expression status of the immunoproteasome may be dependent on disease types and their pathogenic mechanisms. On the other hand, it should be noted that our results on the frequencies of β1i polymorphisms of cancer cell lines derived from different organs are not necessarily reflective of those among these cancer types. This is in part due to the small sample size and the experimental design of our current study. In particular, our experimental design involved the exclusion of several cell lines harboring additional polymorphisms in the genes encoding β1i and/or other immunoproteasome subunits such as β5i in order to minimize potential compounding factors. Our results suggest that the codon 60 polymorphisms are not likely to be responsible for the observed variability in the β1i expression/activity among the tested cell lines. In further validating our findings, it may be important to employ a larger sample size of cancer cell lines or clinical samples derived from the same organs, perhaps comparison of samples with comparable β1i expression levels. Additionally, genetic association studies examining the β1i polymorphism at codon 60 in the context of cancer development and progression are also warranted.

In conclusion, our results demonstrate that the β1i subunit of the immunoproteasome is frequently expressed in colon and pancreatic cancers and possibly in other types of solid cancers. In addition, the genetic polymorphism at codon 60 appears to have no major impact on the expression and catalytic activity of β1i in cancer cells. Taken together, these findings may provide useful insights for the development of immunoproteasome-targeting anti-cancer agents. Additionally, these results exclude codon 60 polymorphic status as a potential factor contributing to variable sensitivity to immunoproteasome inhibitors targeting β1i.

## Supporting Information

Table S1 (DOCX)
