# Supplementary material for: PSMB9 Codon 60 Polymorphisms Have No Impact on the Activity of the Immunoproteasome Catalytic Subunit B1i Expressed in Multiple Types of Solid Cancer
Source: PLoS One. 2013 Sep 9;8(9):e73732. doi: 10.1371/journal.pone.0073732 (PMC3767749; doi:10.1371/journal.pone.0073732)
Supplement: Table S1 — (DOCX) [file pone.0073732.s001.docx]

Table S1. β1i expression and activity levels in the human cancer cell lines carrying different codon 60 genotypes

| **cancer type** | **cell lines** | **β1i expression**  **(%, relative to H23)** | **β1i activity  (hydrolysis rate of Ac-PAL-AMC, RFU/min)** | **Codon 60 genotype** |
| --- | --- | --- | --- | --- |
| lung | H23 | 100 | 194.1 | HH |
|  | H358 | 85.5 | 239.6 | RR |
|  | H460 | 25.6 | 98.1 | RR |
|  | H727 | 9.8 | 48.8 | RR |
|  | H1299 | 14 | 78.6 | RR |
|  |  |  |  |  |
| colon | HCT8 | 4.4 | 30.4 | HH |
|  | DLD1 | 27.9 | 116.3 | HH |
|  | HCT116 | 12.9 | 66.7 | HH |
|  | SW480 | 19.5 | 72.8 | RH |
|  |  |  |  |  |
| prostate | PC3 | 3.1 | 53 | RR |
|  | Du145 | 32.4 | 78.8 | RR |
|  |  |  |  |  |
| pancreatic | BxPc3 | 20.9 | 81 | RR |
|  | Panc1 | 53.6 | 121.9 | RR |
|  | AsPC1 | 131.5 | 239.1 | RR |
|  |  |  |  |  |
| breast | MCF7 | 12.6 | 80.6 | RH |
|  | Hs578T | 68.4 | 120.4 | RR |
|  | MDA-MB-231 | 73.7 | 133.6 | RR |
